# Alcohol and pregnenolone interaction on cerebral arteries through targeting of vascular smooth muscle ${Ca}^{2+}$- and voltage-gated K^+^ channels of big conductance

**DOI:** 10.3389/adar.2023.11735

**Published:** 2023-08-14

**Authors:** Kelsey C. North, Andrew A. Shaw, Luiz Moreira, Anna N. Bukiya, Alex M. Dopico

**Affiliations:** Department Pharmacology, Addiction Science and Toxicology, College of Medicine, The University of Tennessee Health Science Center, Memphis, TN, United States

**Keywords:** alcohol intoxication, MaxiK channel, cerebral arteries, neurosteroids, vascular smooth muscle

## Abstract

Despite the significant number of people who may be taking pregnenolone supplements while drinking alcohol (ethanol), the widely documented cerebrovascular actions of pregnenolone and ethanol, and the critical dependence of cerebrovascular function on cerebral artery diameter, there are no studies addressing the effect of pregnenolone + ethanol in combination on cerebral artery diameter. We investigated this by evaluating the effect of this combination on middle cerebral artery diameter in male and female C57BL/6J mice, both *in vivo* and *in vitro*. The use of de-endothelialized, *in vitro* pressurized middle cerebral artery segments allowed us to conduct a concentration-response study of constriction induced by pregnenolone ± ethanol, in which drug action could be evaluated independently of circulating and endothelial factors. In both male and female animals, pregnenolone at lower concentrations (≤1 μM) was found to synergize with 50 mM ethanol to cause vasoconstriction. In both sexes, this synergism was lost as one or both vasoconstrictors approached their maximally effective concentrations (75 mM and 10 μM for ethanol and pregnenolone, respectively), whether this was evaluated *in vitro* or *in vivo* using a cranial window. Vasoconstriction by pregnenolone + ethanol was abolished by 1 μM paxilline, indicating BK channel involvement. Moreover, cell-free recordings of BK channel activity in cerebral artery myocyte membranes showed that 10 μM pregnenolone and pregnenolone +50 mM ethanol reduced channel activity to an identical extent, suggesting that these drugs inhibit cerebrovascular BK channels *via* a common mechanism or mechanisms. Indeed, pregnenolone was found to disrupt allosteric coupling to ${Ca}^{2+}$-driven gating, as previously reported for ethanol.

## Introduction

Binge drinking is the most common pattern of excessive alcohol consumption in the US [1–3] and thus constitutes a major public health concern. Binge drinking is a pattern of episodic drinking that results in a blood alcohol concentration (BAC) of >0.08% (i.e., >17.4 mM alcohol), which constitutes legal limit of intoxication to drive motor vehicle in most of the US [4, 5]. Binge drinking occurs at all ages: for example, high school students constitute 14% of all binge drinkers [2], while 33% of college students reportedly binge drink between the ages of 21 and 23 years [6, 7]. Binge drinking in adulthood and in the elderly is associated with an increased incidence of cerebrovascular disease, including both ischemic and hemorrhagic strokes [8–10]. Remarkably, a rapid expansion of alcohol use disorders (AUD) is occurring in the population aged 65 and older [11], a group at particular risk for cerebrovascular ischemic conditions.

In turn, pregnenolone (PREG) is a vasoactive neurosteroid that regulates several physiological processes, including growth and differentiation of glial cells and neuronal firing in the developed brain [12]. PREG supplementation is proposed for the treatment of psychological, mental, and substance use disorders, including AUD [12–18]. There are recent studies suggesting that fluctuations in PREG concentration, as a result of either pathophysiological conditions or therapeutic intervention, could impact not only neuronal but also cerebrovascular function [19, 20]. Therefore, there is potential for an expansion of the segment of the human population that may be engaging in simultaneous intake of PREG and alcohol, a combination that will very likely affect brain artery function.

Ethanol (EtOH) at concentrations reached in the blood during binge drinking constricts cerebral arteries in a wide variety of species, including humans, both *in vivo* and *in vitro* [21–28]. This EtOH action is independent of circulating and endothelial factors; instead, it results from inhibition of the ${Ca}^{2+}$- and voltage-gated K^+^ large conductance channels (BK channels) present in cerebrovascular smooth muscle (SM) [29]. This EtOH action is consistent with the well-established facts that BK channel activation and inhibition lead to cerebrovascular SM relaxation and contraction, respectively, and thus, cerebral artery dilation and constriction [30–32]. Cerebrovascular SM BK channels include channel-forming α (cbv1 channel isoform; [33]) and regulatory β1 subunits [32, 34]. The latter are necessary both for inhibition of cerebrovascular SM BK channels and for eventual cerebral artery constriction by EtOH [25]. In particular, the β1 transmembrane domain (TM) 2 serves as an EtOH sensor [35].

Our group has recently documented the fact that PREG, at local and therapeutically relevant concentrations (sub-to low μM), also inhibits cerebrovascular SM BK channels, eventually inducing constriction of cerebral arteries [19]. In contrast to EtOH, these PREG actions do not require β1 subunits; instead, cbv1 channels suffice [19]. While the separate effects of alcohol and PREG on SM BK channels and cerebral artery diameter have been investigated, the effect of concomitant administration of PREG + EtOH on SM BK activity and cerebral function has not been addressed, despite its important epidemiological and public health implications. To address this issue, we here evaluate the effect of *in vivo* and *in vitro* EtOH+/−PREG administration to the middle cerebral artery (MCA), which provides most of the blood flow to the brain and is most commonly affected by neurovascular ischemia and AUD [36–39]. To obtain mechanistic insights, we evaluate EtOH+/−PREG actions on MCA SM BK channel activity in cell-free systems. Our study reveals that PREG at submaximal constrictive concentrations synergizes with EtOH, thus amplifying the MCA constriction induced by EtOH concentrations (50 mM) obtained in the blood during binge drinking. This synergism is lost when both agents are probed at or close to their maximally effective concentrations, which is explained by their shared targeting of allosteric mechanisms that result in disruption of ${Ca}^{2+}$-driven channel gating.

## Materials and methods

### Ethical aspects of the research

The animal care and experimental protocols were reviewed and approved by the IACUC of the University of Tennessee Health Science Center, which is accredited by the Association for Assessment and Accreditation of Laboratory Animal Care.

### *In vivo* measurements of cerebral artery diameter

C57BL/6J mice of both sexes, all 8–12 weeks old, were anesthetized with a mixture of xylazine/ketamine (12/100 mg/kg of weight) and kept anesthetized for the duration of the experiment with subsequent ketamine doses (50 mg/kg of weight) every 15 min or as needed. A catheter was inserted into the internal carotid artery so that experimental drug infusions were directed toward the brain rather than the thoracic cavity. An area of the skull was cleared of tissue and thinned in order to visualize the branching arteries originating from the middle cerebral artery (MCA) on the brain side where the catheter was inserted, above the zygomatic arch, between the ear and eye [27, 40]. The arteries branching out from the MCA were monitored using a Leica MC170 HD microscope with a mounted camera (Leica M125 C) connected to a computer monitor. Drugs were diluted to their final concentration in 0.9% NaCl and administered *via* catheter at 0.1 mL/25 g of mouse weight. Cranial window images before and after drug administration were acquired every 60 s for later analysis; a sample of *n* = 5–6 was acquired for each group (with n representing the number of separate animals).

### *In vitro* measurements of cerebral artery diameter

Male and female C57BL/6J mice, all 8–12 weeks old, were deeply anesthetized with isoflurane *via* inhalation using an open-drop method in a bell jar. Upon losing their response to toe pinch, animals were quickly decapitated with sharp scissors. Resistance-size MCAs (~100 μm in outer diameter) were dissected from the mouse brains. Endothelium was removed by passing an air bubble through the vessel lumen for 90 s [29]. Arterial segments (0.5 cm long) were cannulated at each end, and the artery exterior was continuously perfused with physiologic sodium saline (PSS) of the following composition (mM): 119 NaCl, 4.7 KCl, 1.2 KH_2_PO_4_, 1.6 CaCl_2_, 1.2 MgSO_4_, 0.023 EDTA, 11 glucose, and 24 NaHCO_3_; pH = 7.4, at 35°C–37°C. PSS was continuously bubbled with O_2_/CO_2_/N_2_ at 21/5/74%. Vehicle control (dimethyl sulfoxide; DMSO), PREG, EtOH, or the PREG + EtOH combination were diluted into PSS and perfused over the arterial segment. The artery external wall diameter was measured using the automatic edge-detection function of the IonWizard software package (IonOptix) *via* a Leica MC170 HD microscope with a mounted camera (Leica M125 C) connected to a computer monitor.

### Electrophysiology data acquisition and analysis

For all electrophysiological recordings, whether in mouse cerebral artery myocytes or following heterologous expression of recombinant BK channels in *Xenopus laevis* oocytes, ionic currents were recorded from excised membrane patches in the inside-out (I/O) patch-clamp configuration. Patch-recording electrodes were pulled from glass capillaries and treated as described previously [41]. When filled with high K+ solution (see below for composition of electrode solutions), the vast majority of tip resistances were ~2 MΩ, with a few reaching 5 MΩ. Series resistance was electronically compensated up to 80% by the EPC8 amplifier. In all experiments, whether on myocytes or oocytes, the nominal free $\left[{Ca}^{2+}\right]$ in experimental solutions was calculated with MaxChelator Sliders (Stanford University) and validated experimentally using ${Ca}^{2+}$-selective and reference electrodes [42]. Solutions were applied to the cytosolic side of the patch using an automated, pressurized Octaflow system (ALA Scientific) through a micropipette tip with an internal diameter of 100 μm. Experiments were carried out at room temperature (20°C–22°C). Ionic currents at single-channel resolution were recorded using an EPC8 amplifier (HEKA) at 1 kHz. Data were digitized at 5 kHz using a Digidata 1320A A/D converter and pCLAMP 8.0 (Molecular Devices).

For ionic current recordings from MCA smooth muscle BK channels, cerebral artery myocytes were isolated from adult mouse MCA as described in detail elsewhere [43]. Bath and electrode solutions contained (mM): 130 KCl, 5 EGTA, 1.6 HEDTA, 2.28 MgCl_2_ ([Mg^2+^]_free_ = 1 mM), 15 HEPES; pH 7.4. Free [${Ca}^{2+}$] in the solution (30 μM) was adjusted to the desired value by adding CaCl_2_. An agar bridge with Cl^−^ as the main anion was used as a ground electrode.

For ionic current recordings in *Xenopus laevis* oocytes, isolated oocytes (stages V and VI) were purchased from *Xenopus* 1. Oocytes were defolliculated with forceps under a microscope and stored at 18°C until injection with cbv1-coding cRNA injection. Each oocyte was injected with 23 nL of 40 ng/μL cbv1 cRNA, with patch-clamp recordings being conducted 36–72 h after injection. Immediately before patch recordings, each oocyte was manually freed from its vitelline layer as described [41]. Both bath and electrode solutions contained (mM) 135 K^+^ gluconate, 5 EGTA, 2.28 MgCl_2_, 15 HEPES, and 1.6 HEDTA, pH 7.4. As for solutions used with myocyte experiments, free [${Ca}^{2+}$] in the solution (30 μM) was adjusted to the desired value by adding CaCl_2_. An agar bridge with K^+^ gluconate as the main anion was used as a ground electrode [41]. Two major ${Ca}^{2+}$-dependent gating parameters, i.e., the ${Ca}^{2+}$ dissociation constant (K_d_) and the allosteric factor (C) that couples ${Ca}^{2+}$-binding to channel close-open transitions in absence of stimuli, were estimated from the ${Ca}^{2+}$ dependence of Po at very negative voltages; such estimates have been used previously to study the effect of EtOH on the ${Ca}^{2+}$ gating behavior of cbv1 channels [44]. To do this, we obtained R0, i.e., the NPo ratio in the presence of ${Ca}^{2+}(0.1-100\mu M)$ over the NPo ratio in absence of ${Ca}^{2+}$ in the absence and presence of 10 μM PREG; data were then fitted using the following equation:

$$R_{0}\left(\left[{Ca}^{2+}\right]\right)\;=\frac{{NP}_{0}\left[V,\;\left[{Ca}^{2+}\right]\right]}{{NP}_{0}[V,\;0]}={\left[\frac{1\;+\;KC}{1\;+\;K}\right]}^{4}\;\;={\left[\frac{1\;+\;C\left[{Ca}^{2+}\right]/Kd}{1\;+\;\left[{Ca}^{2+}\right]/Kd}\right]}^{4}$$

### Chemicals

Pregnenolone was purchased from Abcam. Ethanol (200 proof; E7023) and all other chemicals were purchased from Sigma Aldrich. PREG stock solution was prepared in DMSO and diluted into saline, PSS, or patch-clamp bath solution immediately before application to the animal, artery, or membrane patch, respectively. Each animal or pressurized artery was exposed to vehicle, PREG, EtOH, or the PREG + EtOH combination only once in order to avoid any possible receptor (i.e., BK channel) desensitization [45]. Membrane patches were perfused with increasing concentrations of ${Ca}^{2+}$, first in the absence and then in the presence of PREG.

### Data analysis

Analysis was performed by investigators who were blind to experimental group identity. Changes in artery diameter obtained from cranial window experiments were determined using the ImageJ software package (ImageJ 1.52a, downloaded from https://imagej.nih.gov/ij/download.html). Changes in artery diameter *in vitro* were determined using the IonWizard software package (IonOptix). The product of the number of channels in the membrane patch (N) and the individual open probability (Po) was used as an index of channel steady-state activity. NPo was obtained using a built-in function in pCLAMP 8.0 (Molecular Devices).

Statistical analysis was performed using the InStat3.05 software package (GraphPad). Data distributions were checked using a Kolmogorov–Smirnov approach in cases where the number of observations ≥10. For normally distributed data (Gaussian type), the *t*-test was used to test for statistically significant differences between two groups. For data following a non-Gaussian distribution or whose mode of distribution could not be established with certainty (number of observations <10), the statistical methods employed included the Mann–Whitney test for comparisons between two experimental groups, and the Kruskal–Wallis test followed by Dunn’s post-test for comparisons of three or more experimental groups. The threshold for significance was set at *p* < 0.05; group sizes were determined to achieve greater than or equal to 80% power at this significance threshold. Data are reported in the form mean ± SEM. Final data plotting and fitting processes were conducted using the Origin 2020 software package (OriginLab).

## Results

### Pregnenolone and ethanol administered *in vivo* evoke similar constriction of cerebral arteries without displaying synergism

**FIGURE 1:**
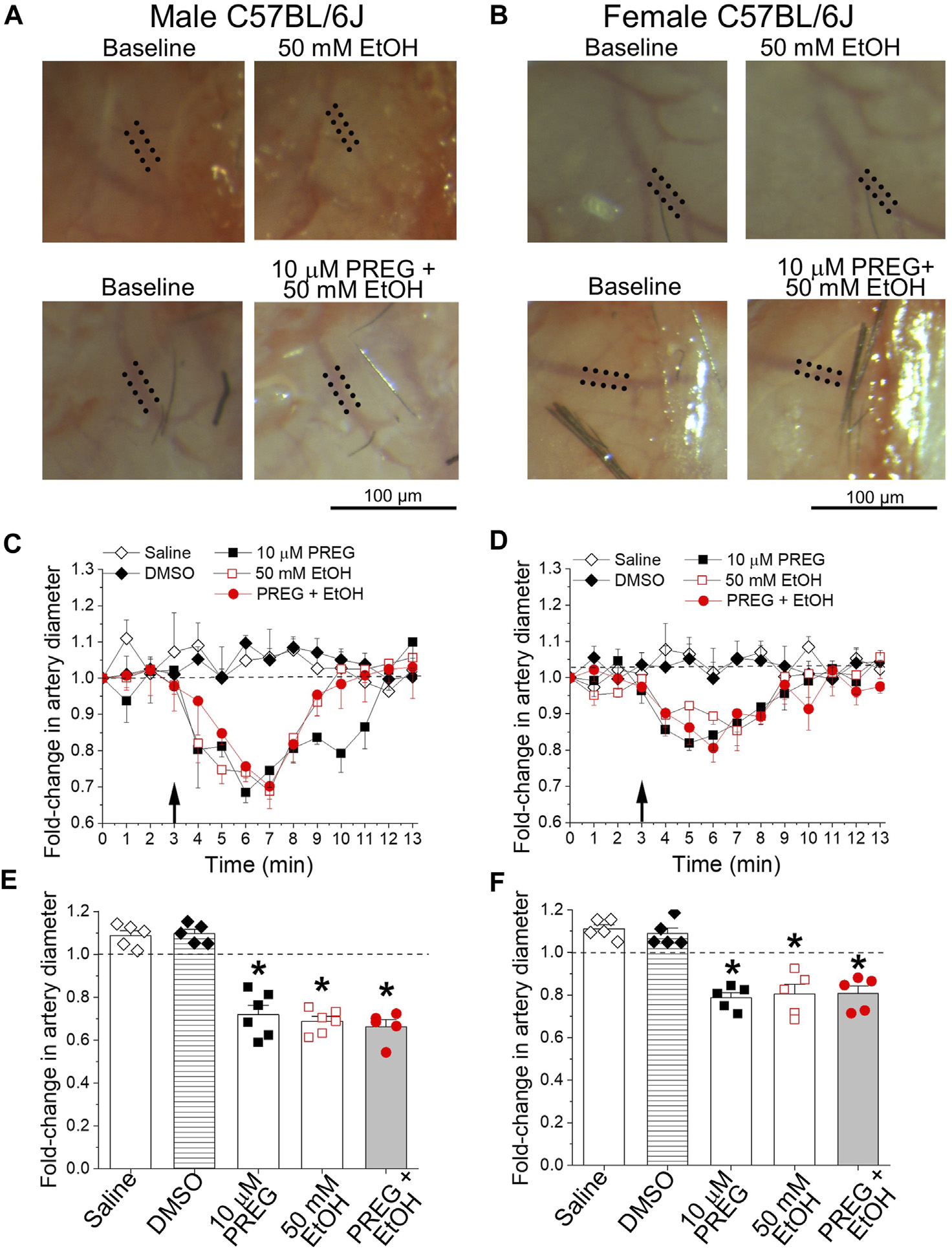
At concentrations known to constrict middle cerebral arteries *in vitro*, pregnenolone and ethanol induced constriction of these arteries *in vivo*, the effects of each agent and their combination being of similar magnitude. **(A)** Representative images showing diameter measurement of pial arteries that branch out of the middle cerebral artery (MCA). Images were obtained *via* a cranial window on male C57BL/6J mice at baseline (pre-drug), and after drug infusion (at 7 min of observation): 50 mM EtOH (top) and the combination of 10 μM PREG with 50 mM EtOH (bottom). Dotted lines highlight outer MCA walls. **(B)** Similar images to those depicted in **(A)**, from age-matched female animals. **(C)** Graph depicting time-dependent changes in pial artery diameter during cranial window recordings for male C57BL/6J mice. The black arrow at minute 3 indicates time of infusion. Here and in **(D–F)**, the horizontal dashed line at 1.0 highlights a lack of effect. **(D)** Graph similar to that shown in **(C)**, showing data from age-matched female animals**. (E)** Average maximal changes in artery diameter from pre-infusion levels compared to volume-matched (saline) and vehicle (DMSO) controls for male mice. **(F)** Graph similar to that shown in **(E)**, showing data from female animals. In both male and female groups: *n* = 5–6/group, where n represents the number of individual mice; *p-value <0.05.

In order to evaluate the combinatory actions of PREG + EtOH on male and female cerebral arteries at the organismal level, we used the cranial window technique. This technique allows for the continuous monitoring of resistance-size pial arteries that branch out of the MCA, and has previously been used to evaluate the pharmacological effects of each drug on these vessels [19, 27]. Intra-carotid infusion of either volume control (0.9% NaCl) or vehicle control (DMSO) failed to evoke significant changes in MCA diameter when compared to averaged pre-infusion values (i.e., the baseline in Figure 1), which were obtained *via* continuous artery diameter monitoring for no less than 3 min. For the testing of PREG, a concentration of 10 μM was chosen because this constitutes the lowest PREG concentration that is able to evoke maximal constriction of the mouse MCA *in vitro* and *in vivo* (EC_max_) [19]. For EtOH, 50 mM was chosen because this concentration is reached in blood circulation after a moderateto-heavy alcohol consumption episode and is close to EC_max_ for ethanol for constriction of mouse cerebral arteries that branch out of Willis’ circle, including the MCA [28]. In contrast to the controls, bolus injections of either PREG or EtOH administered to male animals resulted in 28% ± 3.8% and 31.2% ± 2.1% reductions in artery diameter, respectively (Figure 1). For both agents, maximal constriction was detected around 3 min after bolus injection. The effect of each agent differed significantly from the time-matched effects of either saline or DMSO (*p* = 0.0079–0.0043). In female animals, PREG and EtOH also evoked a peak constriction around 3 min after bolus injection, with arterial diameter decreasing by 21.3% ± 2.2% and 19.5% ± 4.1%, respectively. These vasoconstrictive responses did not differ statistically from those observed in males (Figures 1A, C, E vs. Figures 1B, D, F).

In males and females, concomitant application of PREG + EtOH caused MCA constriction of magnitude 33.6% ± 2.8% and 19.3% ± 3.2%, respectively. Within each sex, the response to the combination PREG + EtOH did not differ statistically from the responses evoked by the individual agents (Figures 1C–F). Since the two agents were applied locally in bolus with the injectate directed toward their site of action (the MCA pial artery branch under recording), the lack of synergism between the PREG and EtOH vasoconstrictive actions is unlikely to have been due to modification of the pharmacokinetic properties (i.e., absorption, distribution, metabolism, and/or elimination) of one drug caused by the simultaneous presence of the other. Rather, the lack of synergism can be explained by (i) the system reaching its maximal level of constriction under each agent and under their combination (a “ceiling effect”), or (ii) convergence of the constrictions elicited by either PREG or EtOH on a given organ/tissue pathway.

### Pregnenolone and ethanol converge on a common pathway to evoke cerebral artery constriction

To investigate the possibility that EtOH and PREG constrict MCA through a common pathway, we used a wide range of PREG concentrations (10 nM–100 μM [19]) in the presence and absence of 50 mM EtOH. If the two cerebrovascular constrictors were acting through a common pathway, then submaximal concentrations of PREG in combination with EtOH at 50 mM would show additivity in constricting the MCA [46]. Since we have previously documented that MCA constriction by either agent does not require the endothelium, but is mediated by targets and mechanisms located in the vascular SM [19, 29], MCA segments were de-endothelialized before pressurization, as described in the Materials and Methods section.

**FIGURE 2:**
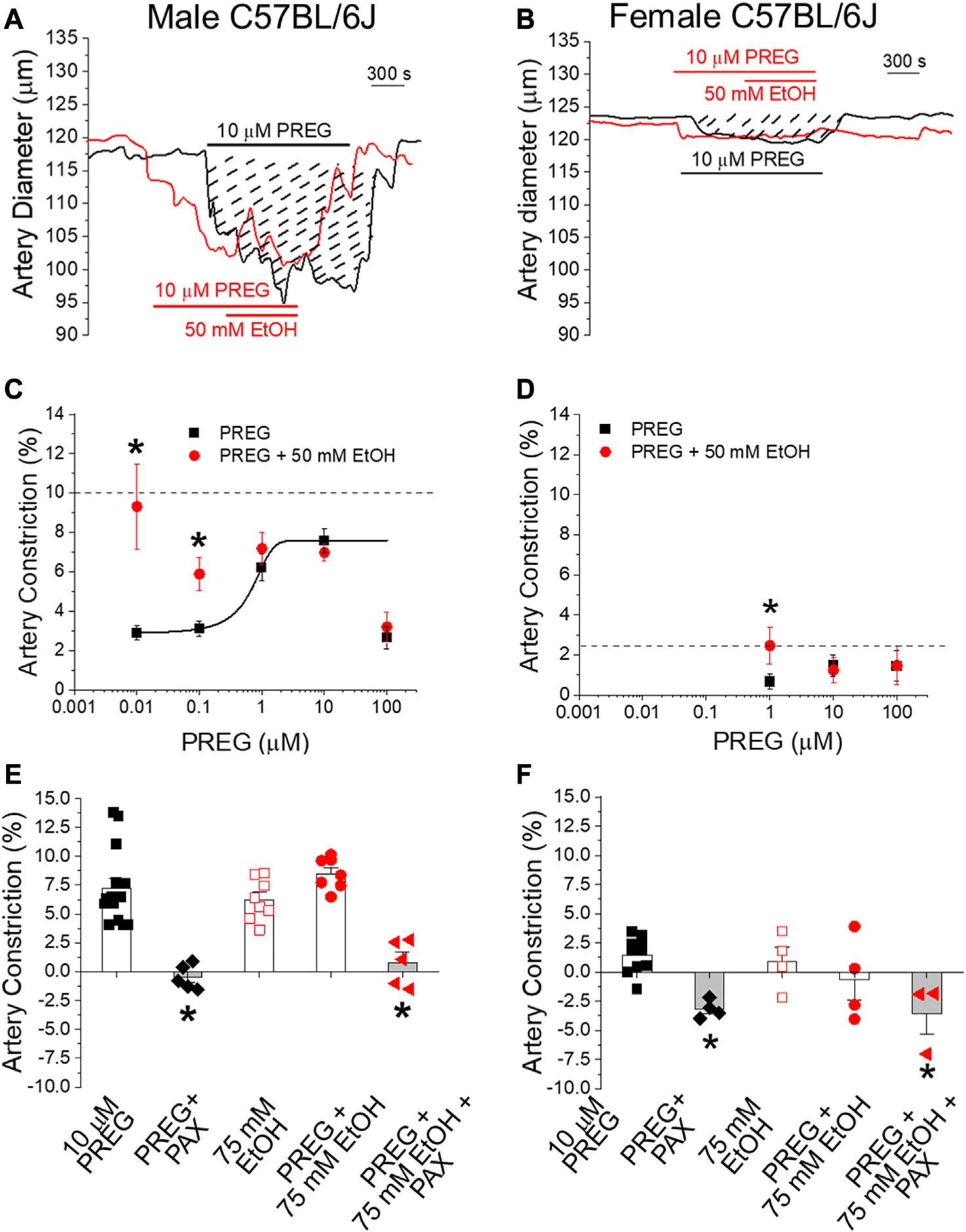
The concentration–response curve for pregnenolone-induced *in vitro* constriction of cerebral arteries in the absence vs. presence of ethanol reveals synergism in the vasoconstrictive effect of these agents at lower pregnenolone concentrations. Matching *in vivo* data, synergism is lost when these ligands reach their maximally effective concentrations. **(A)** Representative traces of time-dependent changes in middle cerebral artery (MCA) diameter for male C57BL/6Jmice. MCA were de-endothelialized and *in vitro* pressurized at 60 mmHg. The black trace shows MCA constriction by 10 μM PREG. Dashed lines highlight the area under the curve, which is indicative of constriction magnitude. The red trace depicts a similar degree of constriction by 10 μM PREG followed by addition of 50 mM EtOH. **(B)** Representative traces of *in vitro* MCA diameter in female animals following manipulations identical to those described for male animals. **(C)** Averaged change in MCA diameter induced by PREG in males. Datapoints for PREG are in black; datapoints for PREG + EtOH are in red. The horizontal dashed line indicates the average constriction evoked by 50 mM EtOH alone. Concentration-dependent constriction by PREG is fitted to a Boltzmann curve; *n* = 6–7 for each PREG concentration. **(D)** Average change in MCA diameter induced by PREG in females, with similar details as provided in the description of the data shown in **(C)**; *n* = 5–7 for each PREG concentration. **(E)** Scatter graphs and average change in MCA diameter in males upon perfusion with the various drugs under investigation and their combinations. **(F)** Average change in MCA diameter in females, with similar details as provided in the description of the data shown in **(E)**. In both (E,F): PAX = 1 μM paxilline; *p < 0.05.

Remarkably, submaximal concentrations (<EC_max_, 0.001–0.1 μM) of PREG that evoke constriction displayed synergism with 50 mM EtOH (Figures 2C, D); in males, the degrees of constriction induced by 10 nM and 100 nM PREG in the presence of co-administrated EtOH (9.32% ± 2.16% and 5.88% ± 0.83% constriction, respectively) were significantly greater than the degrees of constriction produced solely by PREG (2.96% ± 0.38% and 3.31% ± 0.38%); *p* = 0.0159 and *p* = 0.026, respectively. Importantly, this synergism was lost at maximally effective and supramaximal concentrations of PREG (i.e., 1, 10, and 100 μM [19]) (Figure 2C), which is to be expected in a case of two agents acting *via* a common pathway or common target(s).

In females, the concentration–response curve of MCA constriction in response to PREG was restricted to evaluate higher concentrations, shown to be effective in our previous publication [19]. Records from these animals also showed synergism between PREG and EtOH when PREG was probed at submaximal concentrations (1 μM): 2.46% ± 0.91% constriction vs. 0.66% ± 0.38% constriction with PREG alone (*p* = 0.04206; Figure 2D). As found with males, MCA constriction in females under exposure to EtOH and PREG was characterized by loss of synergism when PREG was probed at maximally effective concentrations (10 and 100 μM; [19]) (Figure 2D). Since it has been documented by us and others, both in this system and under identical conditions, that depolarizing 60 mM KCl constricts MCA by >20% in both males and females (see [28] and references cited therein), the lack of synergism between PREG and EtOH in evoking MCA constriction cannot be explained by a “ceiling effect” (i.e., by MCA segments reaching their maximal possible degree of constriction). Therefore, the synergism between EtOH and PREG at submaximal concentrations and the loss of synergism when either EC_Max_ is reached on isolated, de-endothelialized MCA segments indicate that the two drugs converge on a common pathway or target(s), likely located in the vascular SM itself.

Given the involvement of BK channels in EtOH- [25, 28, 29] and PREG-induced [19] constriction of de-endothelialized cerebral arteries, we next probed whether these channels were involved in MCA constriction by PREG or EtOH when these agents were applied in combination vs. separately. Synergism in MCA constriction was not detected either in males or in females when EtOH was probed at 75 mM (Figures 2E, F), extending our findings shown in Figures 2C, D. More importantly, in both males and females, paxilline at a concentration that selectively blocks BK channels (1 μM; [47]) completely abolished the constriction evoked by PREG alone and by PREG + EtOH (Figures 2E, F). This outcome indicates that the common pathway implicated in constriction induced by PREG and EtOH involves SM BK channels.

### BK channel inhibition by pregnenolone and ethanol involves ${Ca}^{2+}$-driven gating

**FIGURE 3:**
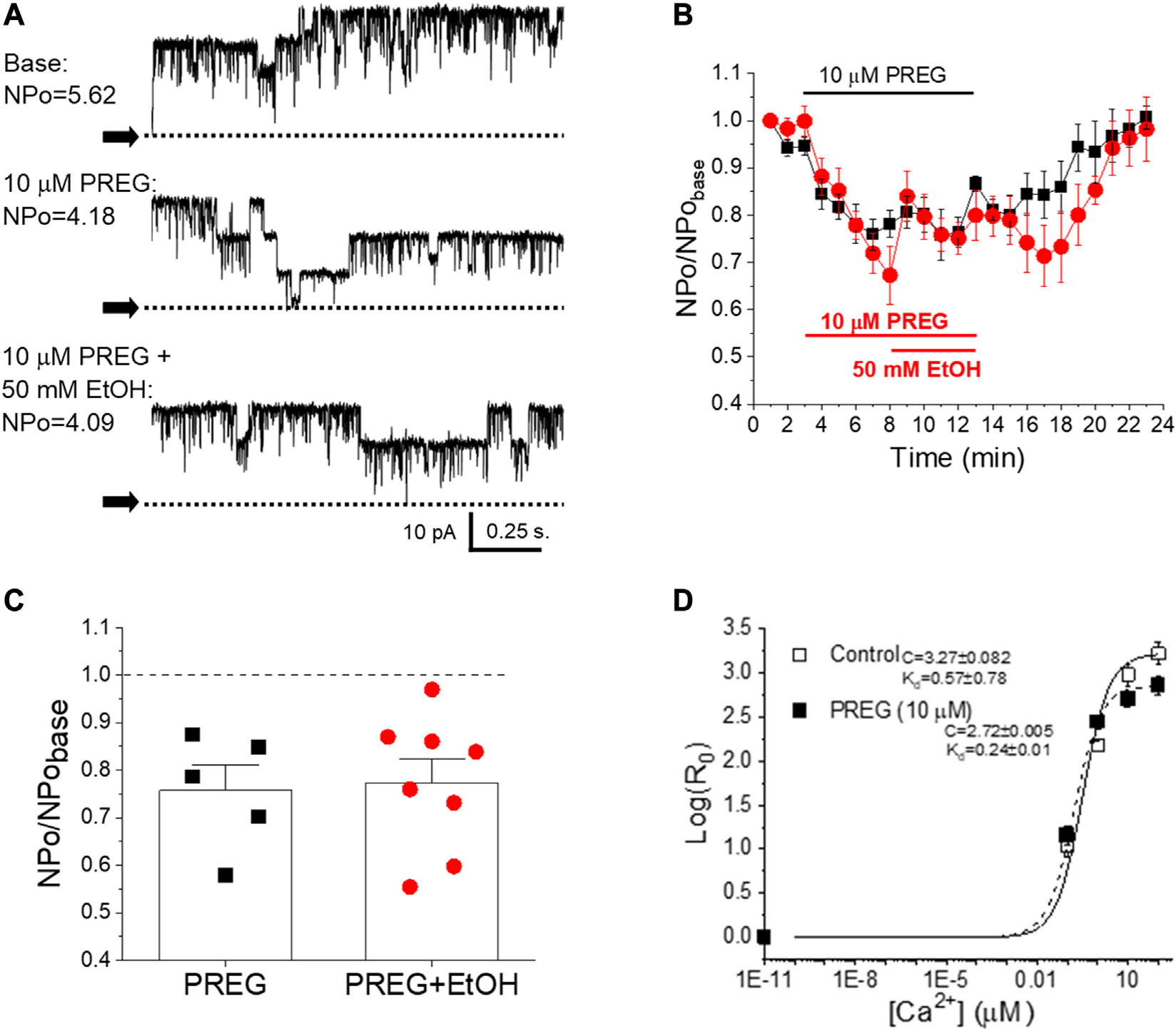
There is no synergism in pregnenolone and ethanol inhibition of BK channels as studied in cell-free systems; both ligands disrupt allosteric coupling to ${{Ca}^{2+}}_{\text{i}}$-driven gating. **(A)** Representative records of BK channel activity (NPo) in inside-out patches from freshly isolated MCA myocytes, obtained before bath patch perfusion with control (top), 10 μM PREG- (middle) and 10 μM PREG+50 mM EtOH-containing bath solutions (bottom); ${\left[{Ca}^{2+}\right]}_{\text{free}}=30\;\text{μ}M$. N = number of channels in the patch; Po = single channel open probability. **(B)** Averaged, time-dependent BK channel inhibition by 10 μM PREG (black) and 10 μM PREG+50 mM EtOH (red). **(C)** Average changes in single datapoints displaying changes in BK NPo induced by PREG vs. PREG + EtOH. A dashed line indicates lack of drug effect. **(D)** Averaged log [R0]-${\left[{Ca}^{2+}\right]}_{\text{i}}$ plots from BK channel-forming cbv1 proteins expressed in *Xenopus laevis* oocytes in the absence and presence of 10 μM PREG, fitted as described in the Material and methods. Best-fit parameters (±95% confidence interval) are shown to the left of the plots. R: NPo ratio in the presence (0.01–100 μM) and absence of ${{Ca}^{2+}}_{\text{i}}$ obtained at 30 mV; Kd: ${Ca}^{2+}$ dissociation constant; C: allosteric parameter coupling ${Ca}^{2+}$-binding to open-to-closed channel transitions in the absence of ${Ca}^{2+}$ or voltage-sensor activation; *n = 3*.

To determine whether SM BK channels were indeed shared targets of EtOH and PREG, we set out to explore whether the lack of synergism in concomitant application of EtOH + PREG could be observed at the level of BK channel activity itself, independently of cell signaling and internal organelles. Thus, we recorded BK channel steady-state activity (NPo) in excised, I/O membrane patches from myocytes freshly isolated from mouse MCA (Figure 3A). We chose 10 μM PREG because this concentration constitutes EC_max_ for PREG-induced constriction of MCA (Figure 2) and for BK channel inhibition by this neurosteroid [19]. The data showed that the inhibition of channel activity by PREG was indistinguishable from that evoked by PREG + EtOH. Indeed, the application of 10 μM PREG to the patch decreased BK NPo to 0.7 ± 0.04 of pre-drug levels (baseline in Figures 3B, C), while the concomitant application of PREG + EtOH decreased NPo to 0.71 ± 0.04 from the baseline (Figures 3B, C). Moreover, the inhibition of BK channel steady-state activity by either 10 μM PREG or 10 μM PREG+50 mM EtOH reported here is identical to the inhibition evoked by 50 mM EtOH alone [25, 29]. Since cerebrovascular SM BK channel inhibition is a well-known mechanism leading to cerebral artery constriction [30, 32], the lack of synergism between EtOH and PREG actions when probed at maximal concentrations in terms of their impact on SM BK channel activity, de-endothelialized MCA segments, and MCA *in vivo* supports the idea that inhibition of cerebrovascular SM BK channels is the common mechanism underlying MCA constriction induced by these drugs (see Discussion).

We have previously documented the finding that EtOH inhibition of BK channels at physiological levels of ${Ca}^{2+}$ found in cerebrovascular myocytes requires the presence of modulatory, smooth muscle-abundant BK β1 subunits [25]. Moreover, the β1 subunit TM2 acts as a specific EtOH sensor [35]. In contrast, PREG-induced inhibition of these channels does not involve β1 regulatory proteins; instead, channel-forming α subunits suffice for steroid action [19]. While each ligand inhibits BK channel activity through recognition by different subunits that form part of the SM BK channel heteromer, the action of the two ligands must converge on a gating mechanism or mechanisms in order to explain their lack of synergism at maximal concentrations (shown in Figure 3). Therefore, we probed the effect of PREG on BK channel currents mediated by recombinant BK channel proteins cloned from cerebrovascular smooth muscle (cbv1 isoform; Material and Methods) and expressed in *Xenopus laevis* oocytes; this system allows for proper comparison with data previously obtained with EtOH under identical recording conditions [44]. Importantly, PREG has been shown to be ineffective in the absence of activating concentrations of ${Ca}^{2+}$ [48]. Therefore, we focused on determining the action of PREG on the ${Ca}^{2+}$-driven gating of cbv1 channels. Specifically, we derived the changes in the channel’s ${Ca}^{2+}$ dissociation constant (K_d_) and the allosteric coupling parameter (i.e., parameter C in the HA model [49]) that links ${Ca}^{2+}$-binding to the intrinsic channel gating (i.e., closed-to-open transitions) occurring in the absence of ${Ca}^{2+}$ binding and membrane depolarization. Both parameters were obtained as described in the Materials and Methods section. Figure 3D shows that 10 μM PREG, surprisingly, did not increase K_d_, but rather decreased it. However, PREG did decrease C, an allosteric decoupling that likely contributes to the inhibitory action of PREG on this channel. Remarkably, these two parameters of cbv1 channel gating are also targeted by EtOH, and the overall effect of this drug on cbv1 channel activity is largely determined by its actions on K_d_ and C [44]. Whether PREG actions on K_d_ and C are the primary determinants of overall PREG-induced inhibition of BK channels remains to be confirmed (*see* Discussion).

## Discussion

Our study provides both translational and mechanistic information on the cerebrovascular effects of two easily accessible drugs: PREG and EtOH. PREG-containing formulations (at 500 mg/day) are proposed as therapeutics against prevalent psychiatric and substance-use disorders, including alcohol misuse [12–19]. In turn, moderate-to-heavy episodic alcohol consumption, e.g., “binge drinking,” which results in BAC around 50 mM EtOH (as used in the current study), constitutes the most prevalent form of alcohol misuse in the US and other developed countries [1–3]. Moreover, approximately 90% of individuals affected by alcohol misuse disorders will relapse within 4 years, according to the National Institute on Alcohol Abuse and Alcoholism [50]. Therefore, mood-stabilizing supplements containing PREG could be frequently consumed by individuals who binge-drink alcohol. Furthermore, the contribution of cerebrovascular ischemia to prevalent disorders is being increasingly recognized. Indeed, alterations in normal control of cerebral artery diameter play a significant role in the pathophysiology of vascular dementia, migraines, seizures, and cerebral vasospasm [51–53]. While (i) the constriction of cerebral arteries by toxicologically relevant concentrations of EtOH has been widely reported in several species, including humans ([28] and references therein), and (ii) the constriction of cerebral arteries by therapeutically relevant concentrations of PREG has been previously reported by our group [19], the current study is the first to determine the effect of PREG combined with EtOH on cerebral artery diameter. The data clearly demonstrate that submaximal vasoconstrictive concentrations of PREG (subμM), i.e., concentrations equivalent to those found in the blood in humans following administration of PREG supplements, are able to potentiate the constriction of cerebral arteries (MCAs) by 50 mM EtOH (Figure 2). Thus, it is reasonable to propose that the ischemic effects of intoxicating levels of alcohol (≤50 mM) will be potentiated by the presence of PREG (sub-to low μM) in brain circulation, and *vice versa*. Regarding the changes in diameter reported here in response to separate or combined administration of EtOH and PREG (i.e., less than 10% decrease from pre-drug administration values), it is important to underscore that even mild changes in cerebral artery diameter are expected to result in robust alterations in brain perfusion, since according to Poiseuille’s law, flow rate is directly proportional to the 4th power of vessel radius [54].

Our study had also documented the finding that, as PREG concentrations and their associated constriction of MCA increased, the synergism with EtOH diminished (Figure 2). Indeed, at concentrations for each ligand that were close to the EC_max_ to evoke MCA (EtOH≥50 mM and PREG≥10 μM), the vasoconstrictive effect of EtOH, PREG, or their combination was similar, whether this was studied *in vivo* through a cranial window (Figure 1) or *in vitro* through isolated MCA segments that had been previously de-endothelialized and pressurized to obtain physiological smooth muscle tone before drug application (Figure 2). These findings are consistent with the involvement of a common mechanism or target in EtOH- and PREG-induced constriction of MCA. The observations that selective channel block by paxilline abolished PREG and EtOH action (Figure 2), and that PREG and EtOH did not show synergism in their inhibitory action on smooth muscle BK channels when studied in free-cell systems (Figure 3), strongly suggest that smooth muscle BK channels themselves are the common effectors of PREG- and EtOH-induced constriction of MCA.

In light of previous findings, our data also constitute important findings from a mechanistic standpoint. On the one hand, it has previously been shown by our group that smooth muscle BK channel inhibition and eventual MCA constriction are dependent on the presence of BK regulatory subunits of β1 type, which are abundant in cerebrovascular smooth muscle [25, 32]. In particular, the TM2 domain of this accessory subunit serves as an alcohol sensor [35]. In contrast, PREG inhibits MCA smooth muscle BK channels and thus evokes constriction *via* its recognition by the channel-forming subunit [19]. Even though EtOH and PREG are directly sensed by different proteins that participate in cerebrovascular SM BK channel heteromers, the lack of inhibitory synergism in their impact on channel activity at maximal concentrations of these drugs (Figure 3) indicates that both modulators must converge on some gating process(es) to inhibit BK channels. Several pieces of evidence support the idea that EtOH and PREG both modulate ${Ca}^{2+}$-driven gating to inhibit cerebrovascular BK channel activity. First, neither EtOH [55] nor PREG [48] changes BK channel activity in the absence of activating (≥1 μM) levels of ${{Ca}^{2+}}_{\text{i}}$, i.e., when the channel is gated by intrinsic activity and/or voltage-sensor activation [44, 48]. Second, mutations that render both high-affinity ${Ca}^{2+}$ sensing domains in the BK channel cytosolic tail domain (CTD) nonfunctional abolish EtOH action on BK channels [55]. In particular, inhibition of homomeric slo1 channels by EtOH requires ${Ca}^{2+}$-activation *via* the high-affinity ${Ca}^{2+}$ site located in the RCK1 [55]. Likewise, CTD deletion [48] or rendering the RCK1 ${Ca}^{2+}$-recognition site nonfunctional *via* the D362A, D367A substitutions abolishes PREG inhibition of cbv1 channel activity [48]. Lastly, both EtOH [44] and PREG (Figure 3D) target ${Ca}^{2+}$-driven parameters of BK channel gating to modify activity. Under similar recording conditions to those used in the present study, EtOH has been found to inhibit BK channels that include β1 subunits [44]. While a minor decrease in K_d_ is caused by exposure of these heteromers to EtOH, this increase in ${Ca}^{2+}$ apparent affinity is overridden by the EtOH-induced decrease in allosteric coupling of ${Ca}^{2+}$-binding to (i) intrinsic gating (i.e., a decrease in parameter C) and (ii) voltage-sensor activation (i.e., a decrease in parameter E) without significant modification of any other voltage-dependent parameter of gating [44]. The current data showed that cbv1 activity was decreased by PREG despite the fact that K_d_ was decreased. Therefore, as previously revealed for EtOH, PREG-induced disruption of allosteric coupling to ${Ca}^{2+}$-gating is the mechanism leading to the overall decrease in channel activity observed when the channel is exposed to PREG. Indeed, our data have revealed that allosteric coupling between ${Ca}^{2+}$-binding and intrinsic channel activity (parameter C) is reduced by PREG (Figure 3D), this action being a key contributor to PREG inhibition of BK channels.

## Data Availability

The original contributions presented in the study are included in the article/supplementary material, further inquiries can be directed to the corresponding author.
